# The impact of serial mergers and acquisitions on enterprises’ total factor productivity: The mediating role of digital transformation

**DOI:** 10.1371/journal.pone.0311045

**Published:** 2024-11-20

**Authors:** Liyan Tan, Xiaoxu Zhang, Yu Song, Fei Zou, Qiaoran Liao

**Affiliations:** School of Business Administration, University of Science and Technology Liaoning, Anshan, Chinae; University of Central Punjab, PAKISTAN

## Abstract

**Research background:**

M&A (Mergers and acquisitions) is a strategic measure for enterprises to expand their scale, enhance their competitiveness and improve productivity in the market competition. As a new factor of production, data is changing the factor input model and value creation path of enterprises.

**Research objectives:**

From the perspective of serial M&A, this study explores the impact of serial M&A on enterprises’ TFP (total factor productivity) and the mechanism of digital transformation between them.

**Research methods:**

Take the serial M&A transactions of China’s A-share listed companies from 2010 to 2019 as samples, using the theory of organizational learning to analyze the relationship among serial M&A, enterprises’ TFP and the degree of digital transformation. Three-step regression is used to construct a model that serial M&A indirectly affects enterprises’ TFP through intermediary variable digital transformation.

**Research finding:**

There is a significant inverse U-shaped relationship between serial M&A and enterprises’ TFP, and digital transformation plays a mediating role in this relationship. The impact of serial M&A on enterprises’ TFP shows an upward trend at first and then a downward trend and this relationship is indirectly realized through digital transformation. The results are still valid after considering the change-explained variables, lag test, Sobel-Goodman test, and Bootstrap test. Heterogeneity analysis shows that for enterprises with non-state-owned property rights, smaller enterprise scale, and higher business environment index, serial M&A has a more obvious effect on TFP indirectly through the degree of digital transformation.

**Research value:**

It further enriches the existing literature on the decision-making of M&A from the perspective of serial M&A and profoundly reveals the mechanism of the degree of digital transformation in the relationship between serial M&A and enterprises’ TFP. The research provides theoretical support and empirical evidence for enterprises to achieve high-quality development.

## 1. Introduction

TFP represents the level of productivity development and is usually used to reflect the input-output efficiency in the macroeconomic field. At present, scholars have gradually introduced the measurement method of TFP into the study of micro-enterprises and studied the factors that affect the enterprises’ TFP from the perspective of enterprises’ management behaviour. In recent years, academia has found that not only the scale of investment in production factors such as labour, capital and the level of technological innovation will affect enterprises’ TFP, but the impact of M&A transactions on productivity has become increasingly obvious [1, 2].As one of the expansion methods for micro-enterprises to quickly obtain resources, M&A not only determine the development strategy and operating boundaries of enterprises but also are an important form of promoting the free flow of production factors [3, 4], which is conducive to the formation of an industry development pattern dominated by high-efficiency enterprises. Studies have shown that M&A is an important economic activity for enterprises to obtain economies of scale, enhance competitive advantages, achieve diversified development, and improve the efficiency of enterprise resource allocation [5, 6], reducing business operating costs and administrative expenses [7–9], improving corporate capital and labour [10], improving technological openness [11] and other aspects are of great significance.

In the study of firm size, operating leverage ratio, and corporate performance, scholars have found that M&A can produce economies of scale, to improve corporate production efficiency and profitability [12, 13]. At the same time, more and more enterprises acquire strategic resources such as technology, brand, and market share through M&A to achieve value creation [11, 14, 15].In this context, enterprises often need to implement corporate strategies through a series of M&A, and a single M&A is often a part of a series of M&A plans. Therefore, in the academic field, multiple M&As completed by the same enterprise within a period is called "serial M&A". Given this, many enterprises are no longer satisfied with the occasional "single M&A", but carry out "serial M&A", such as China’s Byte dance serial M&A cases, Tencent serial M&A cases, and Huawei serial M&A cases, the United States Facebook serial M&A cases, Nike serial M&A cases, Walmart serial M&A cases and the Wall Street Journal serial M&A cases, etc. All of them achieved technology acquisition, technical shortcomings repaired, and business expansion through serial M&A, and achieved significant market position and corporate value enhancement in their respective fields. Then, with the increase of serial M&A transactions in the M&A market, it is of great theoretical value and practical significance to discuss the impact of serial M&A on enterprises’ TFP.

Digital transformation is the reconstruction of the way of value creation driven by digital technology, which facilitates the optimization of organizational structure, manufacturing, marketing, R&D, and product iteration. The study has found that digital transformation significantly enhances the resource base and dynamic capability required by enterprises to deal with crises, and is the key driving force for the improvement of organizational resilience [16–19]. In the context of the digital economy, digital transformation has become a key element for enterprises to enhance competitiveness, adapt to the rapidly changing market environment, and improve enterprises’ TFP. It can be seen that serial M&A and digital transformation will help maximize corporate value and improve enterprises’ TFP. M&A emphasizes the improvement of enterprises’ TFP through synergy effects, while digital transformation focuses on improving the overall operating efficiency of enterprises through digital technology, thereby achieving the improvement of enterprises’ TFP. At the same time, as the number of serial M&A increases, new technologies, professionals or new business models may be brought to the enterprise, which may promote the degree of digital transformation of the enterprise to varying degrees, which in turn will improve corporate value and TFP. The improvement brings a positive promoting effect. Then, in the context of digital transformation, can serial M&A optimize and integrate M&A resources through digital transformation, and then promote the enterprises’ TFP? The answer to this question will help to investigate the impact of serial M&A decisions on the enterprises’ TFP and explore the mechanism and transmission paths.

The research questions of this study are as follows: In this study, the M&A transactions of Chinese listed companies from 2010 to 2019 are selected as the total sample and refer to Schipper and Thompson (1983) [20] select companies that have three or more M&A transactions during the sample period. The empirical research method is used to test the relationship between the number of serial M&A, the degree of digital transformation, and the enterprises’ TFP.

The possible research contributions of this study are as follows: First, it will enrich the research on the impact of serial M&A on the enterprises’ TFP. By studying the impact of the number of serial M&A on the enterprises’ TFP, we can provide an in-depth understanding and explanation of the development quality of serial M&A from both theoretical and empirical perspectives. Through theoretical analysis and systematic empirical research, this study provides best practices for enterprises to make serial M&A decision-making and digital transformation management to achieve better TFP results during the process of serial M&A. Second, it will help to elaborate the mediating effect of digital transformation in the relationship of serial M&A affecting TFP. In the context of the rapid development of the digital economy, the digital transformation of enterprises plays an important role in the relationship between the number of serial M&A and the high-quality development of enterprises. Studying how digital transformation plays a mediating role in the impact of serial M&A on the quality of corporate development will help academics to understand the mechanism of digital transformation’ work on integration effects, resource optimization, and business innovation. This will guide business management decisions to better utilize digital transformation to promote serial M&A decisions and TFP. Third, we further explore the relationship between the number of serial M&A, the degree of digital transformation, and the enterprises’ TFP from the perspective of property rights, firm size, and business environment index. The findings of this study will help to enrich the key factors affecting the TFP of serial M&A enterprises. It is of great significance how to regulate and guide the M&A decisions of enterprises to enhance their TFP for different situations, and provides empirical evidence for the formulation of relevant policies.

The remainder of this study is organized as follows: Section 2 is the literature review, which, summarizes the existing research and expounds on the similarities and differences between this study and the existing research; Section 3 is the theoretical analysis and research hypothesis, which expounds the relationship between serial M&A and enterprise TFP, as well as the mediating effect of digital transformation; Section 4 is research design, which introduces sample selection, variable description, and model design. Section 5 is the empirical analysis and discussion, which carries on the empirical test to the research hypothesis, and carries on the result analysis and discussion; Section 6 is the conclusion.

## 2. Literature review

Based on the driving factors of M&A efficiency, some scholars have shown that the more enterprises pay attention to resource integration activities, the easier they are to realize resource synergies [21]. The studies of Ahuja and Katila (2001), Anderson et al. (2015), and Popli et al. (2017) have shown that, with the introduction of new technical knowledge, management, and marketing experience, M&A enterprises can promote the synergistic effect of resources of both M&A parties [22–24] and help enterprises create value [24]. At the same time, M&A enables enterprises to apply their technical knowledge, management and marketing experience to more markets, which helps to improve the efficiency of resource allocation, all of which can enhance the enterprises’ productivity [25]. When both parties of M&A are better matched in terms of similarity and complementarity of resources, the greater the possibility of achieving synergies through economies of scale and scope [26, 27]. Given this, many enterprises are more willing to realize efficiency improvement and high-quality development with the help of serial M&A.

Based on the economic consequences of M&A. Some scholars have shown that M&A can significantly contribute to the TFP of both parties of M&A [1, 2], and can improve the efficiency of M&A [10, 25, 28–30] and economic benefits of enterprises [31, 32]. However, M&A enterprises may also reduce efficiency due to the increase in organizational costs [33]. Some scholars have also found that M&A significantly reduce corporate value [34, 35]. Scholars have proposed the theory of inefficiency motivation for M&A [36], which mainly includes the motivation of managers to build the Empire State Building [37–39] and managerial ego [40, 41] et al. For their benefit, managers build the "Empire State Building" [42], engage in affiliate M&A, transfer benefits, and hollow out listed companies [43]. In addition, too much M&A reflects the agency costs of the acquirer’s management [44]. Managers may also be overconfident [45], and overestimate the benefits of M&A and M&A projects with low returns on investment, thus damaging corporate value [46]. Bertrand and Betschinger (2012) [47] based on the analysis of more than 600 samples of M&A in Russia, which is shown that the problems brought by M&A, such as agency problems, new resource integration and organizational costs will lead to the decline of corporate performance. Aybar and Ficici (2009) [48] have studied 433 overseas M&A transactions of 58 multinational companies in emerging economies from 1991 to 2004 and found that M&A not only failed to create value but also actually damaged corporate performance. Some scholars believe that the impact of mergers and acquisitions on enterprises’ TFP is uncertain. Gort and Nakil (1999) [49] studied the impact of M&A on the productivity and operating costs of two domestic telecommunications companies in the United States from 1997 to 1998.The results have showed that there were no significant differences in TFP before and after the M&A, but instead increased operating costs. Amess (2003) [50] has taken the British companies that had management buyouts (MBOs) during 1986–1997 as samples and applied the Cobb-Douglas production function to explore the productivity effect of these companies. The empirical results have found that the TFP of those enterprises adopting the MBO governance structure increased by 16.13% on average. The marginal value of labour has also increased, but the marginal value of capital has decreased.

In addition, some scholars believe that there may be a more complex nonlinear relationship between M&A and the economic consequences of enterprises. According to the contingency theory, Haleblian and Finkelstein (1999) [51] have believed that only when enterprises accumulate sufficient experience can they distinguish the economic performance of a current M&A from the economic performance of a previous M&A. Therefore, they believe that the M&A experience has a positive U-shaped relationship with M&A performance. Zollo and Reuer (2010) [52] have studied the relationship between domestic M&A experience and performance of American commercial banking industry, and drew a positive U-shaped conclusion. Nadolska and Barkema (2014) [53] have analyzed the cross-border M&A of 1038 Dutch enterprises and found that there is a positive U-shaped relationship between experience and performance. Some scholars also believe that there is no significant relationship between M&A experience and M&A performance, or there is an inverted U-shaped relationship. For example, Zollo and Singh (2004) [54] have took U.S. domestic M&A as samples and draw the conclusion that there is no significant relationship between experience and performance. Kroll et al. (1997) [55] has studied the large-scale M&A in the mining and manufacturing industries in the United States and also concluded that there is no significant relationship. Hayward (2002) [56] has measured experience through the similarity between the focal M&A and previous M&A experience. If the previous M&A experience is highly similar, and there is a lack of M&A skills applicable to all M&A. However, if the previous M&A experience is highly different, then there is a lack of M&A skills applicable to any M&A. Therefore, the high similarity or difference between previous M&A experience and the low level of focal M&A performance indicates an inverted U-shaped relationship between M&A experience and M&A performance.

Through the review of the above literature, it can be seen that the conclusions of the existing literature on M&A value creation mainly focus on the positive impact, negative impact, uncertainty impact and nonlinear impact. From the perspective of measurement indicators of economic consequences, most of the studies on this issue focus on the impact of M&A on financial performance, while there are few studies on the impact of TFP. At the same time, existing studies mainly focus on the impact of a single M&A on corporate value, that is to say, behind most M&A literature is an assumption that regards each M&A transaction as an independent event and ignores the difference between a single M&A and serial M&A [57–60]. Although there are not many directly related literature, the relevant literature provide useful references for this study. Based on the theories of organizational learning, this study will focus on the impact of serial mergers and acquisitions on the total factor productivity of enterprises and the mediating role of digital transformation on the relationship between the two.

## 3. Theoretical analysis and research hypothesis

### 3.1. Serial M&A and enterprise’s TFP

According to organizational learning theory, the impact of serial M&A on enterprises’ TFP can be discussed from two aspects: M&A experience and M&A inertia. First, with the increase in the number of serial M&A, companies gradually accumulate M&A experience through "learning by doing" [61] and gain competitive advantages through serial M&A [62, 63], thereby improving the enterprises’ TFP. At the same time, as M&A experience increases, corporate managers may over-rely on past M&A experience and practices, resulting in a decline in corporate investment capabilities, conservative management, and uneven allocation of serial M&A resources, thus hindering the improvement of enterprises’ TFP. Therefore, through the accumulation of experience and knowledge, M&A experience can bring synergy effects to M&A companies, optimize resource allocation, reduce redundancy, improve efficiency, and have a positive impact on the enterprises’ TFP. M&A inertia may make the enterprises’ serial M&A objectives relatively monotonous, including M&A involving similar or overlapping markets and fields, reducing the efficiency of optimal allocation of corporate resources, and thus hurting the enterprises’ TFP.

First, the impact of M&A experience on enterprises’ TFP. As the number of serial M&A increases, M&A experience gradually accumulates, companies ’ability to learn M&A knowledge continues to be strengthened, and useful information is extracted from experience information for their subsequent M&A behaviour. Organizational learning theory holds that experiential learning is the process by which an organization acquires, understands, disseminates, expands, and applies its experience [64]. Enterprises accumulate experience in past behaviour. As experience accumulates, they gradually accumulate relevant experience and knowledge of M&A integration and M&A management [65]. This kind of experience and knowledge accumulation can bring synergies to M&A enterprises, and have a positive impact on the enterprises’ TFP by optimizing resource allocation, reducing redundancy, and improving efficiency.

Second, the impact of M&A inertia on enterprises’ TFP. Whether M&A experience can have a positive impact on the overall productivity of an enterprise depends on the enterprise’s absorption and application of M&A experience. When an enterprise has M&A inertia due to the accumulation of experience and knowledge, serial M&A is not necessarily conducive to the development of the enterprise. Enterprises may draw wrong inferences from past M&A or use these inferences in inappropriate ways. To maintain a competitive advantage, enterprises need to have unique resources and capabilities that are difficult to imitate or replace. However, due to M&A inertia, enterprises may show greater similarities in M&A target preferences. Although this imitation strategy has the advantages of reducing M&A risks and reducing M&A costs, with the increase in the number of serial M&A, the organizational structure and resource integration scale of the enterprise become more and more complex, which may cause cultural conflicts, lag in information transmission, problems such as hindered knowledge sharing and improper resource allocation make it difficult for enterprises to achieve their development advantages. In turn, it may harm the improvement of enterprises’ TFP.

To sum up, with the increase in the number of serial M&A, the enterprises’ TFP may show a trend of increasing first and then decreasing. In the early stages of serial M&A, enterprises are in the stage of organizational learning and adaptation. With the accumulation of M&A experience and the transfer of knowledge, the enterprises’ TFP will improve. However, with the increase in the number of serial M&A, corporate learning gradually reaches a saturation point, and the weakening of the learning effect causes serial M&A enterprises to fall into the inertia of imitating previous M&A strategies. Based on the above views, this study believes that the M&A experience of organizational learning can bring synergy effects to enterprises in the early stage of serial M&A and improve the enterprises’ TFP. With the increase in the number of serial M&A, enterprises have experienced M&A inertia, and previous experience and knowledge have gradually devalued. The difficulty of M&A integration and organizational inertia hinder the improvement of enterprises’ TFP. Therefore, this study proposes the following hypothesis:

Hypothesis 1: There is a significant inverse U-shaped relationship between serial M&A and TFP.

### 3.2. The mediating effect of digital transformation

Studies have shown that digital transformation is the key for enterprises to obtain long-term competitive advantages [66]. With the help of digital transformation, enterprises can seek a more dynamic development mode [67], which provides the possibility for enterprises to improve overall productivity. The implementation of serial M&A enables enterprises to have scarce resources that are not easy to imitate [68], so that enterprises can quickly acquire advanced digital technology and management experience, and provide support for promoting enterprises’ digital transformation. Through serial M&A, enterprises can integrate the M&A objects with digital technology and digital transformation experience, and obtain advanced digital technology, digital expertise and digital platform. These digital transformation capabilities can help serial M&A enterprises deal with complex business after M&A integration, optimize the M&A integration process, and improve the efficiency of resource integration. As a special intangible asset, digital technology, digital expertise, and digital platforms are characterized by high external transaction costs, high risks, and rapid technological changes. Through M&A, enterprises can control digital technology, digital expertise, and digital platforms in a short period, helping enterprises achieve digital transformation [69]. The enhancement of digital transformation capabilities allows enterprises to respond to market changes quickly, allowing serial M&A enterprises to quickly adapt to market changes, seize market opportunities, and thereby improve their TFP. At the same time, serial M&A can also prevent competitors from acquiring key digital expertise and establish a technical "moat" [70]. After the M&A, the inherent dynamism and extensibility of digital technology will also promote the recombination of corporate knowledge, and the establishment of digital knowledge bases and derivative innovation [71–73], to help M&A enterprises to achieve synergies in production, operation, finance, talent and technology, and thus promote the improvement of total factor productivity of enterprises.

The synergy of technology and knowledge creates direct conditions for the acquired enterprises to improve production efficiency, so it may improve TFP. From the perspective of empirical research, through technology and knowledge sharing, the acquired enterprises can master more advanced technologies, to improve production efficiency [5]. Digital transformation can help enterprises promote knowledge sharing and collaboration between both parties, thereby improving the enterprises’ TFP. In the process of digital transformation, M&A enterprises can optimize resource allocation and capital structure, increase the spillover effects of resources and capital, and promote the improvement of enterprises’ TFP. At the same time, there may be a non-linear relationship between the number of serial M&A and the degree of digital transformation. As the number of serial M&As increases, enterprises may experience a learning curve when it comes to digital transformation. In the initial phase of M&A, enterprises may need to adapt to new digital technologies and business processes and overcome integration barriers. With the accumulation and learning of experience, enterprises can cope with the challenges of serial M&A and digital transformation, and improve the effectiveness of digital transformation. However, as the number of serial M&A increases, the speed of the learning curve may slow down, leading to a decline in the effectiveness of digital transformation and inhibiting the improvement of enterprises’ TFP. Therefore, to explore the mediating effect of digital transformation between the number of serial M&A and the enterprises’ TFP, this study proposes the following hypothesis.

Hypothesis 2: Enterprise digital transformation plays a mediating role between serial M&A and TFP.

**Fig 1:**
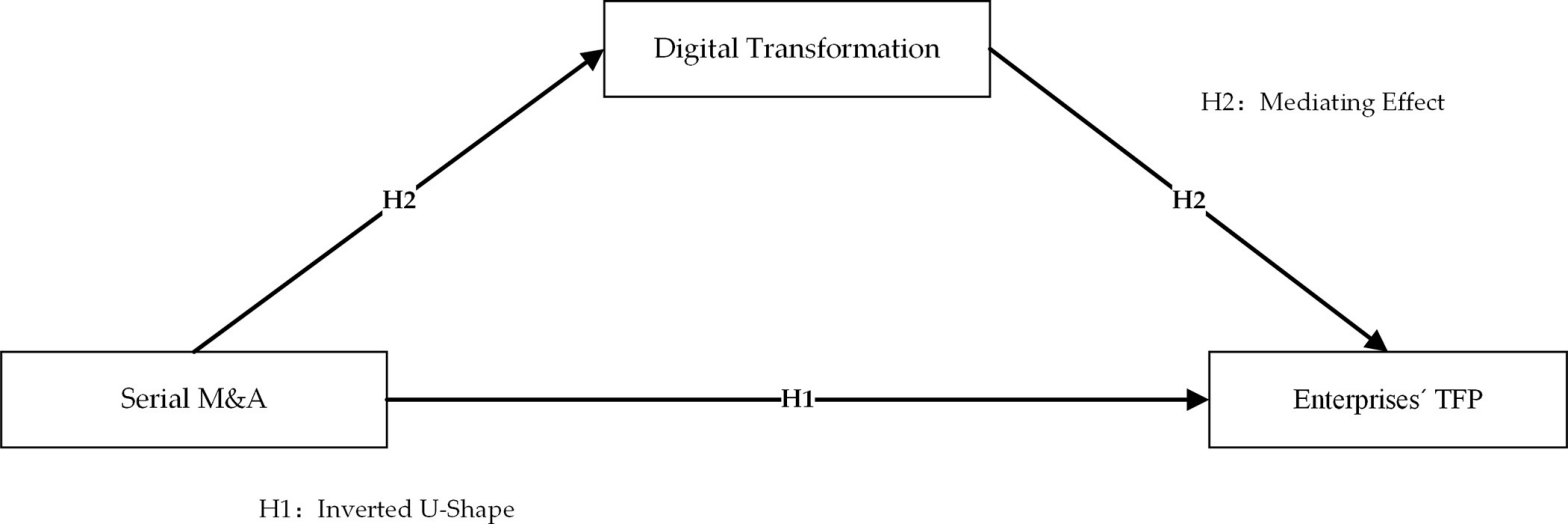
Theoretical model.

The theoretical model is shown in Fig 1.

## 4. Research design

### 4.1. Sample selection

This study focuses on M&A activities within Chinese A-share listed companies from 2010 to 2019 and selects the focal companies according to engage in at least three M&A transactions in the sample period. The sample data are processed as follows: (1) The failed or incomplete samples of M&A are excluded; (2) If a company has multiple M&A on the date of the first merger announcement, only the sample of M&A with the largest transaction value will be retained; (3) Excluding the samples which M&A transaction amount is missing or less than 1 million yuan; (4) Removing affiliate M&A transaction samples; (5) Excluding financial and insurance samples; (6) Remove ST, ST* and PT samples; (7) Filtering out samples with asset-liability ratio below 0 or above 1; (8) Removing missing samples with lacking financial data. After the above screening, the total observed values of 12 367 samples were obtained.

This study chooses 2010–2019 as the sample interval because this interval is representative. Since this study focuses on the enterprises’ TFP and takes into account the lag effects of preconditions such as serial M&A and digital transformation have a certain time lag, we do not consider the data after the outbreak of COVID-19. In this study, 2019 is chosen as the termination year of the research sample. On the other hand, the China Digital Economy Development Report (2022) shows that the scale of digital transformation of Chinese enterprises has been growing since 2010, which has become a new driving force to promote the high-quality development of enterprises, so this study chooses 2010 as the starting year of the research sample. The construction of total factor productivity of listed companies and other microdata at the enterprise level comes from the China Stock Market & Accounting Research Database (CSMAR). The data processing mainly adopts STATA14.0. Research data can be available in S1 Data.

### 4.2. Variable description

#### 4.2.1. Explained variable: Enterprise’s TFP (*TFP*_*i*,*t*_*_LP*)

Productivity is the key index to evaluate production efficiency, which can be divided into single-factor productivity and TFP. Single-factor productivity measures the ratio of total economic output to the amount of input to a factor of production, while TFP is the remaining part of the output growth rate after deducting the output of labour, capital, and other factors. According to Solow (1957), this remaining part is mainly caused by technological progress [74]. At the macro level, TFP reflects the proportion of total national output to various input factors and represents the efficiency of resource utilization in the whole society. At the micro level, the enterprises’ TFP represents the upgrading of technology and the improvement of production efficiency, which plays an important role in the accounting of production efficiency, and is also one of the important manifestations of enterprise heterogeneity. this study focuses on the measurement of TFP from the micro level. By referring to the practice of Krishnan, Nandy and Puri (2015) [75], this study estimates the logarithmic Cobb-Douglas production function and obtains the measurement index of TFP (*TFP*_*i*,*t*__*LP*) of listed companies.


$$LnY_{i,t}=\chi_{0}+\chi_{1}LnK_{i,t}+\chi_{2}LnL_{i,t}+\chi_{3}LnM_{i,t}+wt+\eta t$$ (1)

In model (1), where Y represents the operating income, K represents the capital input, using the net value of fixed assets of the enterprise, L represents the labour input, expressed by the number of employees of the listed company, M represents the intermediate input, measured by the "cash paid for goods and services" by the enterprise. Directly regressing the residuals of Model (1) using OLS would introduce bias into the calculation of TFP, while the semi-parametric method can better overcome problems such as simultaneous bias and selective bias. Therefore, in this study, the LP semi-parametric method is used to calculate the enterprises’ TFP [76], and the results obtained from the OP semi-parametric method are used to conduct the robustness test of alternative variables.

#### 4.2.2. Explanatory variable: Serial M&A(*SMA*_*i*,*t*_)

The number of M&A completed by the sample serial M&A enterprise *i* in year *t* is measured.

#### 4.2.3. Mediating variable: Digital transformation (*DCG*_*i*,*t*_)

**Fig 2:**
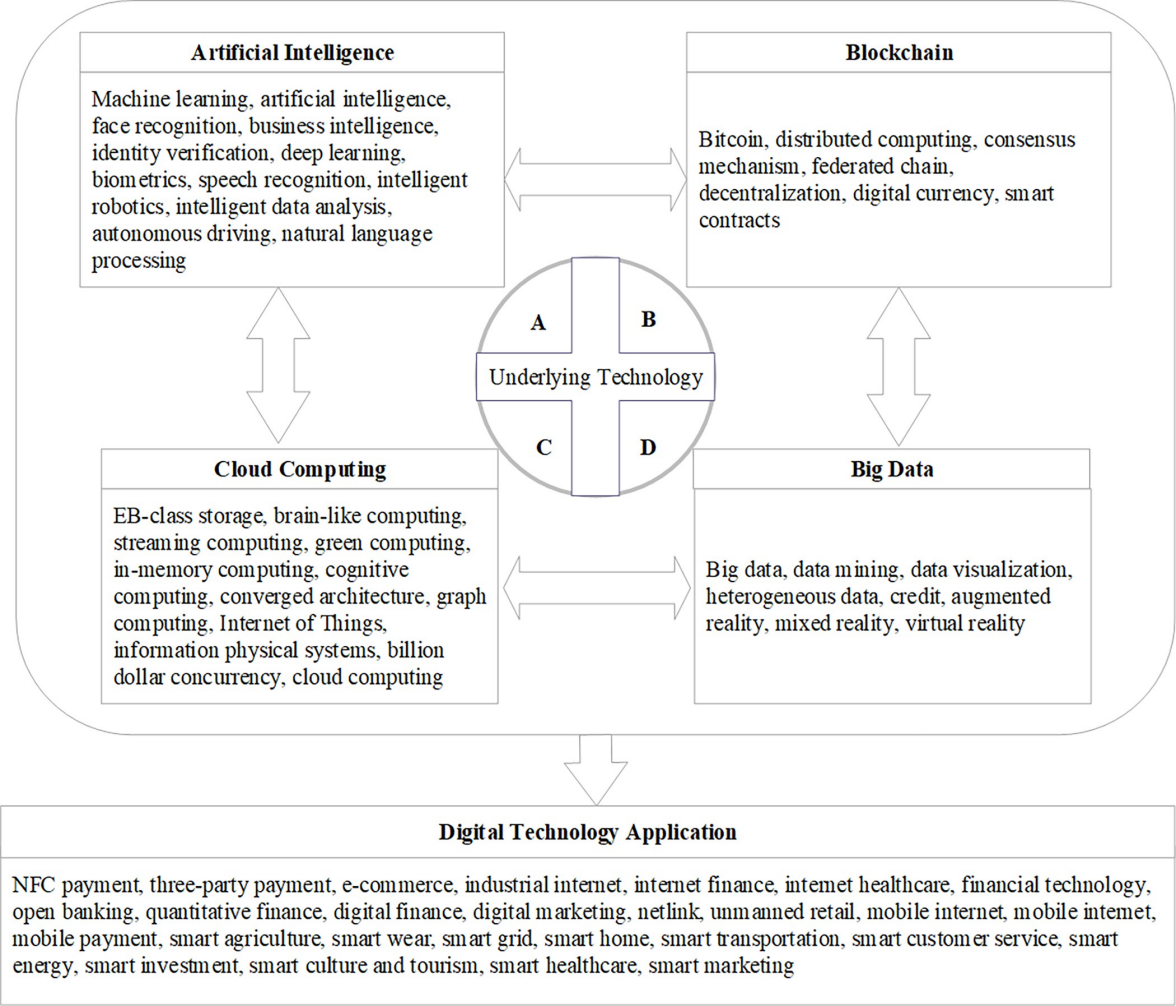
Keyword map for digital transformation.

At present, the measurement methods of digital transformation mainly include quantitative description and text analysis. The quantitative description method is mainly measured by the proportion of the digital technology-related items in the intangible assets detailed in the notes to the financial report to the total intangible assets. The rule of text analysis is to use big data technology intelligent analysis to measure the frequency of keywords of digital transformation in financial reports. The corporate digital transformation discussed in this study focuses on the research and application of artificial intelligence, blockchain, cloud computing, big data and other new-generation information technology of listed companies in China, so compared with quantitative description, text analysis is more in line with the original intention of this study. On the one hand, by screening the intangible assets detailed items that meet certain conditions in the notes to the annual financial statements, it is difficult to effectively cut into the R&D and application of the new generation of information technology, and in a sense, there are more obvious measurement errors of variable indicators. On the other hand, mining the text information of enterprise annual financial reports by using the increasingly mature computer intelligent analysis function is helpful to achieve the research purpose of multi-dimensional and accurate and effective measurement of digital transformation. Therefore, this study adopts text analysis to measure digital transformation. Based on the practice of Wu, Hu, and Lin et al. (2021) [77], this study uses the Python crawler function to classify five indicators of artificial intelligence technology, blockchain technology, cloud computing technology, big data technology, and digital technology application and summarizes and sorts out the feature word frequency related to digital transformation in the annual financial report of enterprises. The keyword map of the specific digital transformation and its matching and summary are shown in Fig 2. The greater the number of characteristic words in digital transformation, the higher the degree of digital transformation. In addition, since the word frequency distribution of the feature words of digital transformation has a statistical tendency to the right, this study adds 1 to the word frequency data for logarithmic processing.

#### 4.2.4. Control variable

Referring to previous studies by Krishnan, Nandy, and Puri (2015) [75], this study mainly controls firm size, firm leverage, Tobin’s Q, director independence, board size, ownership concentration, property rights, and business environment index. The definitions of the variables are described in Table 1.

**Table 1:** Definition of main variables.

*Type*	*Name*	*Symbol*	*Definition*
*Explained variable*	*TFP*	*TFP*_*i*,*t*__*LP*	The total factor production efficiency of enterprises is calculated by the LP method.
*Explanatory variable*	*Serial M&A*	*SMA*_*i*,*t*_	The number of M&A of enterprise *i* in t.
*Mediating variable*	*Digital transformation*	*DCG*_*i*,*t*_	Digital transformation feature words frequently add 1 to take the logarithm
*Control variables*	*Firm size*	*Size*_*i*,*t*_	Add 1 to the total assets
	*Firm leverage*	*Lev*_*i*,*t*_	Liabilities as a percentage of total assets
	*Tobin’s Q*	*TobQ*_*i*,*t*_	The ratio of the market value of the enterprise to the replacement cost of assets
	*Board independence*	*Indep*_*i*,*t*_	The percentage of independent directors to total number of directors
	*Board size*	*Board*_*i*,*t*_	Total number of directors on the board
	*Ownership concentration*	*Share*_*i*,*t*_	The percentage of the largest shareholder
	*Property rights*	*State*_*i*,*t*_	The dummy variable, equal to 1 if the enterprise ownership is state-owned, 0 otherwise.
	*Business environment index*	*Envir*_*i*,*t*_	The dummy variable, equal to 1 if the business environment index of the province (CN) is high, 0 otherwise.

### 4.3. Model design

Using the mediating effect test method of Baron and Kenny (1986) for reference, this study constructs a recursive model to test the impact of serial M&A on the enterprises’ TFP, as well as the mediating role of digital transformation. Considering the nonlinear relationship between serial M&A and enterprises’ TFP, this study puts the primary and secondary variables of serial M&A into the model at the same time. To make the test results comparable, the control variables are kept consistent. Based on the analysis of hypothesis 1 above, the benchmark model is set as follows:

$$TFP_{i,t}\_LP=\alpha_{0}+\alpha_{1}SMA_{i,t}^{2}+\alpha_{2}SMA_{i,t}+\sum \alpha_{i}Control_{i,t}+\sum industry+\sum Year+\varepsilon$$ (2)

Where, *α*_*0*_ is the constant term; *α*_*i*_(*i* = 1,2,…,8) represents the estimated coefficient of the model; The explained variable *TFP*_*i*,*t*__*LP* represents the TFP of serial M&A enterprise *i* in year *t*. The explanatory variable represents the number of M&A completed by serial M&A enterprise *i* in year *t*; The control variables represent the set of control variables in Table 1; *Industry* represents the fixed effect of the industry; *Year* represents the annual fixed effect; *ε* represents the random error term. *α*_1_ of the model is the focused object, which is used to investigate the inverted U-shaped influence of serial M&A on the TFP of enterprises. If hypothesis 1 is valid, *α*_1_<0 and significant.

Drawing on the practice of Baron and Kenny (1986) [78], Zhang, Song and Liu (2023) [60], based on model (2), digital transformation (*DCG*_*i*,*t*_) with intermediary variables is introduced, and the model is set as follows:

$$DCG_{i,t}=\beta_{0}+\beta_{1}SMA_{i,t}^{2}+\beta_{2}SMA_{i,t}+\sum \beta_{i}Control_{i,t}+\sum industry+\sum Year+\varepsilon$$ (3)

$$TFP_{i,t}\_LP=\chi_{0}+\chi_{1}SMA_{i,t}^{2}+\chi_{2}SMA_{i,t}+\chi_{3}DCG_{i,t}+\sum \chi_{i}Control_{i,t}+\sum industry+\sum Year+\varepsilon$$ (4)

If the quadratic estimation coefficient of serial M&A in the model (3) is significant and *β*_*1*_<0, it indicates that there is a significant inverse U-shaped relationship between serial M&A and the degree of digital transformation. If the estimated coefficient of the degree of enterprise’s digital transformation in model (4) is significant and *χ*_3_>0, the absolute values of the coefficients of the primary and quadratic coefficients of serial M&A become smaller or less significant, indicating that the degree of digital transformation plays a mediating effect between serial M&A and TFP.

## 5. Empirical analysis and discussion

### 5.1. Descriptive statistics and correlation analysis

#### 5.1.1. Sample characteristics of serial M&A

**Fig 3:**
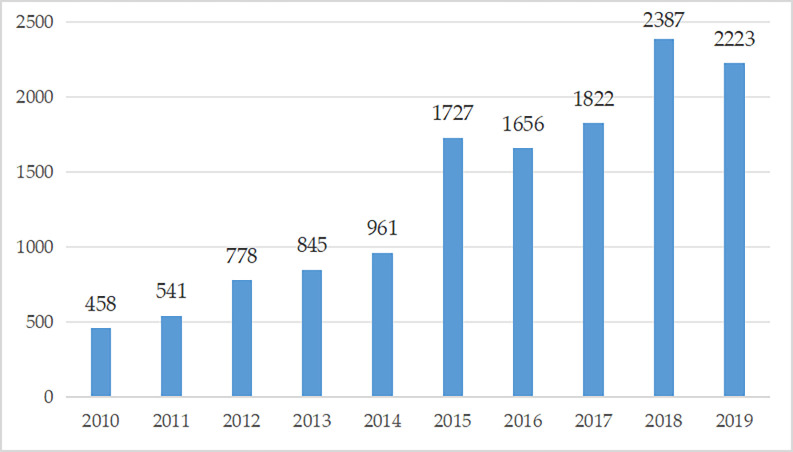
Number of serial M&As of listed companies in China from 2010 to 2019.

Fig 3 shows the number of serial M&A transactions of Chinese A-share listed companies from 2010 to 2019. From 2010 to 2015, the number of serial M&A of Chinese A-share listed companies increased year by year. In 2010, there are only 458 serial M&A transactions, and in 2015, there were 1,727 serial M&As, an increase of about four times. Due to the complex and volatile capital market environment, the number of serial M&A transactions decreased slightly in 2016, but there are still 1,656 transactions. The number of serial M&A have rose again in 2017, peaking in 2018 and declining slightly in 2019. In general, the number of serial M&A of Chinese A-share listed companies showed a tortuous upward trend from 2010 to 2019.

#### 5.1.2. Descriptive statistics and correlation analysis

In Table 1, columns (2) and (3) report the mean value and standard deviation of the main variables. Among them, the mean value and standard deviation of the enterprises’ TFP (*TFP*_*i*,*t*__*lp*) are 6.896 and 3.307, indicating that there is a significant gap in the TFP of Chinese A-share serial M&A from 2010 to 2019. The mean and standard deviation of the number of serial M&A (*SMA*_*i*,*t*_) of enterprises are 1.242 and 1.909, indicating that the average number of M&A of sample enterprises is no less than 1 times per year. The mean value of enterprise digital transformation (*DCG*_*i*,*t*_) is 0.355, and the standard deviation is 0.683, indicating that there is also a certain gap in the digital transformation of sample enterprises. The descriptive statistics of other variables are consistent with the existing literature.

In this study, Pearson correlation analysis is used to conduct a preliminary test on the relationship between variables. As can be seen from Table 2, there is a significant positive correlation between the annual number of serial M&A and the enterprises’ TFP, and the correlation coefficient is 0.088, p<0.001, indicating that the more serial M&A, the higher the enterprises’ TFP. Hypothesis 1 has preliminary support for the left side. There is a significant positive correlation between the degree of enterprise digital transformation and TFP (correlation coefficient 0.091, p<0.001) and the number of serial M&A (correlation coefficient 0.284, p<0.001), respectively. However, the intermediary effect of enterprise digital transformation still needs to be further tested.

**Table 2:** Descriptive statistics and correlations of main variables.

Variables	1	2	3	4	5	6	7	8	9	10	11
*1*.*TFP*_*i*,*t*_*_lp*	1										
*2*.*SMA*_*i*,*t*_	0.088***	1									
*3*.*DCG*_*i*,*t*_	0.091***	0.284***	1								
*4*.*Size*_*i*,*t*_	0.293***	0.095***	0.112***	1							
*5*.*Lev*_*i*,*t*_	0.041***	0.030***	-0.005	0.318***	1						
*6*.*Tobin′Q*_*i*,*t*_	-0.057***	-0.008	-0.009	-0.160***	-0.032***	1					
*7*.*Indep*_*i*,*t*_	0.023**	0.015	0.035***	0.029***	0.012	0.017*	1				
*8*.*Board*_*i*,*t*_	0.046***	-0.040***	-0.017*	0.297***	0.113***	-0.051***	-0.345***	1			
*9*.*ShareTop*_*i*,*t*_	0.136***	-0.051***	-0.056***	0.226***	0.016*	-0.038***	0.019**	0.012	1		
*10*.*State*_*i*,*t*_	0.108***	-0.189***	-0.116***	0.218***	0.147***	-0.035***	-0.017*	0.188***	0.189***	1	
*11*.*Envir*_*i*,*t*_	-0.031***	0.040***	0.043***	0.046***	-0.072***	0.006	-0.018**	0.002	0.063***	-0.043***	1
*mean*	6.896	1.242	0.355	22.393	0.509	2.523	0.372	8.864	0.341	0.506	0.609
*S*.*d*.	3.307	1.909	0.683	1.525	0.223	18.910	0.058	1.898	0.153	0.500	0.488

Notes: The correlation analysis shown in the table is the Pearson test.

*p<0.1

**p<0.05

***ap<0.01

### 5.2. Empirical results and analysis

#### 5.2.1. U-test and benchmark analysis

Table 3 shows the U-test of the influence of on *TFP*_*i*,*t*__*lp*. The result shows that the value ranges from 0 to 40, and the extreme point is 12.076. The results of the P value show that there is a significant correlation between variables. There is a significant positive correlation between variables in the range from 0 to the lower limit. In the lower limit of the interval to 40, there has significant negative correlation between variables. The change of slope indicates that there is a significant nonlinear relationship between variables. At the same time, the slope has a negative sign in the interval, and it can be considered that the relationship between *SMA*_*i*,*t*_ and *TFP*_*i*,*t*__*lp* is inverted "U" type. Hypothesis 1 is tentatively supported. Column (1) in Table 4 shows the benchmark regression analysis results of the impact of serial M&A on the enterprises’ TFP. The results show that the second item *SMA*_*i*,*t*_^2^ and the first item *SMA*_*i*,*t*_ of serial M&A are significantly correlated with the TFP (*TFP*_*i*,*t*__*lp*) at a 1% level. Among them, the estimated coefficient *SMA*_*i*,*t*_^2^ is -0.005, and that of is 0.178, indicating that the number of serial M&A has an inverted U-shaped influence on the enterprises’ TFP. Hypothesis 1 is supported.

**Table 3:** U-test for testing non-linearity.

	*Interval*	*Slope*	*t-value*	*p-value*
*Lower Bound*	0	0.086	6.362	0.000
*Upper Bound*	40	−0.198	-3.029	0.001

**Table 4:** The regression results of the models.

*Variable*	(1)	(2)	(3)
	*TFP*_*i*,*t*__*lp*	*DCG*_*i*,*t*_	*TFP*_*i*,*t*__*lp*
*SMA*_*i*,*t*_^2^	-0.005***	-0.004***	-0.004***
	(-5.13)	(-11.10)	(-4.30)
*SMA*_*i*,*t*_	0.178***	0.119***	0.144***
	(9.25)	(22.19)	(7.38)
*DCG*_*i*,*t*_	-	-	0.281***
			(7.26)
*Size*_*i*,*t*_	0.882***	0.044***	0.869***
	(37.65)	(9.48)	(37.08)
*Leverage*_*i*,*t*_	-1.123***	0.011	-1.126***
	(-7.80)	(0.42)	(-7.84)
*Tobin′Q*_*i*,*t*_	0.0004	-0.0002	0.0004
	(0.74)	(-1.43)	(0.86)
*Indep*_*i*,*t*_	-0.435	0.208**	-0.493
	(-0.87)	(2.11)	(-0.99)
*Board*_*i*,*t*_	-0.044***	-0.004	-0.043**
	(-2.58)	(-1.19)	(-2.52)
*Firstshare*_*i*,*t*_	0.637***	-0.132***	0.674***
	(3.22)	(-3.31)	(3.42)
*State*_*i*,*t*_	0.450***	-0.059***	0.467***
	(7.71)	(-4.67)	(7.98)
*Envir*_*i*,*t*_	-0.392***	0.168	-0.396***
	(-7.48)	(1.42)	(-7.59)
*Industry & Year*	*Control*	*Control*	*Control*
*Constant*	-11.667***	-0.963***	-11.396***
	(-22.58)	(-9.76)	(-22.03)
*Observation*	12 367	12 367	12 367
*R* ^*2*^	0.260 1	0.181 7	0.262 9

Note: t-values are in parentheses.

*p<0.1

**p<0.05

***p<0.01.

#### 5.2.2. Mediating effect of digital transformation

As can be seen from column (2) of Table 4, the estimated coefficient *SMA*_*i*,*t*_^2^ is -0.004, and that of *SMA*_*i*,*t*_ is 0.119, both of which are significant at the 1% level, indicating an inverted U-shaped relationship between serial M&A and the degree of digital transformation of enterprises. By observing column (3) of Table 4, it is found that the estimated coefficient of digital transformation *DCG*_*i*,*t*_ is 0.281, which is significant at the 1% level. The estimated coefficient *SMA*_*i*,*t*_ becomes smaller compared to column (1), and the absolute value of the estimated coefficient *SMA*_*i*,*t*_^2^ becomes smaller compared to column (1). It shows that the degree of digital transformation plays a mediating role in the relationship between serial M&A and TFP. Hypothesis 2 is supported.

### 5.3. Robustness test

#### 5.3.1. Replace the measure of total factor productivity

To ensure the robustness of the research results, this study uses the research method of Krishan, Nandy and Puri (2015) [75] for reference, and further adopts the OP semi-parameter method to measure the enterprises’ TFP. Table 5 lists the regression results of measuring the enterprises’ TFP by the OP semi-parameter method. The results in column (1) show that the quadratic coefficient of serial M&A is significant at the 5% level, indicating that serial M&A has a significant inverted U-shaped effect on the enterprises’ TFP, supporting hypothesis 1. The results of column (2) show that the quadratic coefficient of serial M&A is significant at the 1% level; the results of column (3) show that the quadratic coefficient of serial M&A is significant at the 10% level, which is less significant than that of column (1); and the digital transformation coefficient is significant at the 1% level, indicating that digital transformation has a significant intermediary effect between serial M&A and the enterprises’ TFP.

**Table 5:** Results of replacing *TFP*_*i*,*t*_ by OP semi-parametric method.

*Variable*	(1)	(2)	(3)
	*TFT*_*i*,*t*__*op*	*DCG*_*i*,*t*_	*TFP*_*i*,*t*__*op*
*SMA*_*i*,*t*_^2^	-0.001**	-0.004***	-0.001*
	(-2.49)	(-11.10)	(-1.74)
*SMA*_*i*,*t*_	0.015**	0.120***	0.008
	(2.22)	(22.19)	(1.14)
*DCG*_*i*,*t*_	-	-	0.060***
			(3.79)
*Controls& Industry & Year*	*Control*	*Control*	*Control*
*Constant*	-1.124***	-0.963***	-1.066***
	(-5.88)	(-9.76)	(-5.57)
*Observation*	12 367	12 367	12 367
*R* ^*2*^	0.285 2	0.181 7	0.286 1

#### 5.3.2. Test taking into account lag effect

Enterprise’s TFP and digital transformation have a certain time lag, given this, Table 6 adopts the method of one-period lag treatment for all explanatory variables to conduct a robustness test, that is, to test the effect of the explained variables in the current period on the explanatory variables in the previous period. Moreover, when examining the impact of serial M&A on the digital transformation of enterprises, the regression between the digital transformation of enterprises one period behind and the number of serial M&A in the current period is used. The estimated coefficients of *SMA*_*i*,*t*_^2^ of columns (1) and (2) in Table 6 are-0.0059 and-0.004, respectively. The estimated coefficient of *DCG*_*i*,*t*_ of column (3) is 0.031, which is significant at 1% level, and the estimated coefficient of *SMA*_*i*,*t*_^2^ is less than column (1).The results show that the coefficient sign and significance of explanatory variables are consistent with the above, so the test considering the hysteresis effect supports the above hypothesis.

**Table 6:** Results of the lagged period test.

*Variable*	(1)	(2)	(3)
	*TFP*_*i*,*t*__*lp*	*DCG*_*i*,*t*_	*TFP*_*i*,*t*__*lp*
*SMA*_*i*,*t*−1_^2^	-0.0059***	-0.004***	-0.0057***
	(-4.15)	(-2.83)	(-4.05)
*SMA*_*i*,*t*−1_	0.166***	0.164***	0.161***
	(7.41)	(3.99)	(7.19)
*DCG*_*i*,*t*−1_	-	-	0.031***
			(5.22)
*Controls& Industry & Year*	*Control*	*Control*	*Control*
*Constant*	-10.939***	-4.047***	-10.813***
	(-19.93)	(-5.71)	(-19.70)
*Observation*	11 302	11 302	11 302
*R* ^*2*^	0.256 1	0.059 6	0.257 7

#### 5.3.3. Sobel-Goodman test

The Sobel-Goodman method is used to test whether the intermediary effect of enterprise digital transformation is established. Based on the above results, this study takes the enterprises’ TFP as individual explanatory variables and the serial M&A of enterprises as explanatory variables to test the mediating effect of enterprises’ digital transformation. Sobel-Goodman test results show that the P value is less than 0.01, indicating that the intermediary effect of enterprise digital transformation is established. The test results show that enterprise digital transformation accounts for 2.38% of the total effect of serial M&A on the enterprises’ TFP, which verifies the mediating effect of enterprise digital transformation.

#### 5.3.4. Bootstrap test

In Table 7, the Bootstrap method was adopted and the number of self-extraction repetitions was 1000 times to test whether the mediating effect of digital transformation was established. The combination shows that digital transformation significantly mediates serial M&A and the enterprises’ TFP. Within a 95% confidence interval, the lower limit and upper limit of the intermediary test of digital transformation are 0.0160 and 0.0316, and the interval does not contain 0, indicating that the intermediary effect is effective. Among them, the indirect effect accounted for 2.38% and the direct effect accounted for 11.01%.

**Table 7:** Results of Bootstrap test.

*Mediation path*	*Coef*	*SE*	*Z*	*p-value*	*LowerCI*	*UpperCI*
$SMA_{i,t}\to TFP_{i,t}\_lp\to DCG_{i,t}$	0.0238	0.0040	5.98	0.000	0.0160	0.0316
	0.1101	0.0145	7.61	0.000	0.0817	0.1384

#### 5.3.5. Omitted variable test

To attenuate the endogenous problems caused by the deviation of omitted variables, the control variables affecting enterprises’ TFP such as return on assets, CEO duality, executive compensation and R&D investment, were included based on the benchmark model. The test results are shown in Table 8. The research conclusions are consistent with Table 4. The estimated coefficient of *SMA*_*i*,*t*_^2^ of column (1) is -0.005 and significant at a 1% level, which shows that serial M&A have a significant inverted U-shaped effect on enterprises’ TFP, which supports hypothesis 1. The results of columns (1) to (3) indicate that the impact of serial M&A on the enterprises’ TFP is indirectly generated through the digital transformation of the mediator, which supports hypothesis 2.

**Table 8:** Results of the omitted variable test.

*Variable*	(1)	(2)	(3)
	*TFP*_*i*,*t*__*lp*	*DCG*_*i*,*t*_	*TFP*_*i*,*t*__*lp*
*SMA*_*i*,*t*_^2^	-0.005***	-0.004***	-0.004***
	(-5.12)	(-11.17)	(-4.29)
*SMA*_*i*,*t*_	0.172***	0.118***	0.140***
	(9.01)	(22.10)	(7.19)
*DCG*_*i*,*t*_	-	-	0.275***
			(7.09)
*Size*_*i*,*t*_	0.837***	0.040***	0.826***
	(33.38)	(8.28)	(32.95)
*Leverage*_*i*,*t*_	-1.050***	0.380	-1.060***
	(-7.22)	(1.46)	(-7.32)
*Tobin′Q*_*i*,*t*_	0.0002	-0.0001	0.0003
	(0.50)	(-1.12)	(0.59)
*Indep*_*i*,*t*_	-0.343	0.215**	-0.402
	(-0.69)	(2.18)	(-0.81)
*Board*_*i*,*t*_	-0.051***	-0.004	-0.049***
	(-3.00)	(-1.33)	(-2.93)
*Firstshare*_*i*,*t*_	0.687***	-0.131***	0.724***
	(3.49)	(-3.27)	(3.69)
*State*_*i*,*t*_	0.420***	-0.059***	0.436***
	(7.21)	(-4.65)	(7.47)
*Envir*_*i*,*t*_	-0.411***	0.011	-0.414***
	(-7.85)	(0.95)	(-7.93)
*ROA*_*i*,*t*_	4.92e-11**	1.15e-12***	1.75e-11
	(2.54)	(24.90)	(0.88)
*Dual*_*i*,*t*_	-0.247***	-0.024*	-0.240***
	(-3.74)	(-1.67)	(-3.64)
*Salary*_*i*,*t*_	0.142***	0.007	0.140***
	(4.58)	(1.38)	(4.56)
*RD*_*i*,*t*_	0.355	1.170***	0.033
	(0.50)	(3.82)	(0.05)
*Industry & Year*	*Control*	*Control*	*Control*
*Constant*	-12.579***	-0.983***	-12.308***
	(-22.52)	(-9.31)	(-22.03)
*Observation*	12367	12367	12367
*R* ^*2*^	0.2630	0.1847	0.2656

### 5.4. Heterogeneity analysis

#### 5.4.1. Property right heterogeneity

Property rights attribute is one of the important characteristics of listed companies in China. Enterprises with different property rights have obvious differences in government intervention and policy burden, so the relationship between the number of serial M&A and the enterprises’ TFP may also be different. The samples were divided into two groups according to property rights, as shown in Table 9. The results show that in non-state-owned enterprises, there is a significant inverse U-shaped relationship between the number of serial M&A, the degree of digital transformation, and the enterprises’ TFP. In state-owned enterprises, there is no significant correlation between serial M&A and the enterprises’ TFP. To sum up, with the increase of the number of serial M&A, enterprises with non-state-owned property rights are more likely to have an inverted U-shaped impact on the enterprises’ TFP indirectly through the degree of digital transformation.

**Table 9:** Results of property right heterogeneity test.

*Variable*	(1)	(2)	(3)	(4)	(5)	(6)
	*State*_*i*,*t*_ = 0			*State*_*i*,*t*_ = 1		
	*TFP*_*i*,*t*__*lp*	*DCG*_*i*,*t*_	*TFP*_*i*,*t*__*lp*	*TFP*_*i*,*t*__*lp*	*DCG*_*i*,*t*_	*TFP*_*i*,*t*__*lp*
*SMA*_*i*,*t*_^2^	-0.008***	-0.004***	-0.006***	-0.002	-0.005*	-0.002
	(-5.05)	(-10.26)	(-4.26)	(-0.71)	(-1.75)	(-0.58)
*SMA*_*i*,*t*_	0.234***	0.122***	0.186***	0.050	0.114***	0.038
	(9.40)	(17.56)	(7.41)	(1.32)	(7.92)	(1.00)
*DCG*_*i*,*t*_	-	-	0.396***	-	-	0.103
			(8.56)			(1.57)
*Controls& Industry & Year*	*Control*	*Control*	*Control*	*Control*	*Control*	*Control*
*Constant*	-14.278***	-1.332***	-13.751***	-8.106***	-0.757***	-8.028***
	(-17.84)	(-8.17)	(-17.19)	(-10.50)	(-5.34)	(-10.38)
*Observation*	6 105	6 105	6 105	6 262	6 262	6 262
*R* ^*2*^	0.278 0	0.187 2	0.284 1	0.241 9	0.159 0	0.242 3

#### 5.4.2. Firm size heterogeneity

Previous studies have shown that even a small difference in firm size will have a significant impact on the relationship between explanatory variables and explained variables in the model [79]. Considering the criticality of the enterprise size index, to avoid selectivity bias, the sample is divided into two groups according to the average enterprise size, as shown in Table 10. The results show that: In the enterprise size group, there is a significant inverted U-shaped relationship and intermediary effect between the number of serial M&A, the degree of digital transformation, and the enterprises’ TFP. In large-scale enterprises, there is no significant nonlinear relationship between the number of serial M&A and the enterprises’ TFP. To sum up, with the increase in the number of serial M&A, enterprises with relatively small scale are more likely to have an inverted "U-shaped" impact on the enterprises’ TFP indirectly through the degree of digital transformation.

**Table 10:** Results of firm size heterogeneity test.

*Variable*	(1)	(2)	(3)	(4)	(5)	(6)
	*Firm size below the average*			*Firm size above the average*		
	*TFP*_*i*,*t*__*lp*	*DCG*_*i*,*t*_	*TFP*_*i*,*t*__*lp*	*TFP*_*i*,*t*__*lp*	*DCG*_*i*,*t*_	*TFP*_*i*,*t*__*lp*
*SMA*_*i*,*t*_^2^	-0.018***	-0.007***	-0.015***	-0.001	-0.004***	0.0002
	(-3.58)	(-3.09)	(-3.22)	(-0.69)	(-10.96)	(0.25)
*SMA*_*i*,*t*_	0.350***	0.133***	0.292***	0.073***	0.122***	0.046*
	(7.99)	(10.19)	(6.75)	(2.90)	(16.28)	(1.75)
*DCG*_*i*,*t*_	-	-	0.437***	-	-	0.227***
			(7.30)			(4.31)
*Controls& Industry & Year*	*Control*	*Control*	*Control*	*Control*	*Control*	*Control*
*Constant*	4.745***	-0.138*	4.805***	5.193***	-0.147	5.226***
	(9.05)	(-1.85)	(9.19)	(9.53)	(-1.25)	(9.59)
*Observation*	6 554	6 554	6 554	5 813	5 813	5 813
*R* ^*2*^	0.086 4	0.164 8	0.092 5	0.263 6	0.189 6	0.265 7

#### 5.4.3. Business environment index heterogeneity

Studies have found that market-based environmental regulation [80], government environmental attention [81], credit policy [82, 83], and corporate environmental governance [84] have important impacts on enterprise value. The business environment index of the region where the M&A firm is located will also affect the impact of serial M&A on enterprises’ TFP. The impact of serial M&A on the enterprises’ TFP located in regions with higher business environment indexes may be more significant. Based on this, according to the business environment index constructed by Wang et al. (2013) [85], this study divides enterprises into a higher group of business environment index and a lower group of business environment index according to whether the business environment index of the region where the enterprises are located is higher than the national average. The results in Table 11 show that in both scenarios, there is an inverted U-shaped relationship between serial M&A and enterprises’ TFP, and digital transformation plays an intermediary effect in the relationship. The above relationship is more significant when the business environment index is higher. It can be seen that the optimization of the business environment can force serial M&A to affect TFP through digital transformation.

**Table 11:** Results of business environment index heterogeneity test.

*Variable*	(1)	(2)	(3)	(4)	(5)	(6)
	*Envir*_*i*,*t*_ = 0			*Envir*_*i*,*t*_ = 1		
	*TFP*_*i*,*t*__*lp*	*DCG*_*i*,*t*_	*TFP*_*i*,*t*__*lp*	*TFP*_*i*,*t*__*lp*	*DCG*_*i*,*t*_	*TFP*_*i*,*t*__*lp*
*SMA*_*i*,*t*_^2^	-0.008**	-0.006**	-0.006**	-0.006***	-0.004***	-0.005***
	(-2.33)	(-1.96)	(-2.00)	(-5.09)	(-11.13)	(-4.32)
*SMA*_*i*,*t*_	0.137***	0.120***	0.110***	0.205***	0.124***	0.167***
	(3.75)	(7.08)	(2.97)	(8.34)	(18.51)	(6.65)
*DCG*_*i*,*t*_	-	-	0.232***	-	-	0.303***
			(4.25)			(5.83)
*Controls& Industry & Year*	*Control*	*Control*	*Control*	*Control*	*Control*	*Control*
*Constant*	-13.560***	-1.222***	-13.277***	-10.982***	-0.761***	-10.751***
	(-17.13)	(-7.59)	(-16.65)	(-15.12)	(-5.69)	(-14.83)
*Observation*	4 835	4 835	4 835	7 532	7 532	7 532
*R* ^*2*^	0.320 0	0.191 5	0.322 0	0.240 9	0.180 5	0.244 0

## 6. Discussion and conclusion

### 6.1. Research findings

The economic consequences of M&A have always been the focus and hot spot in the field of corporate finance research. However, most M&A studies regard M&A transactions as independent events, and few studies are conducted from the perspective of serial M&A. As a new economic behaviour of the digital economy, digital transformation plays an increasingly prominent role in the enterprises’ TFP. However, at present, there is no research to further test the mechanism of the relationship between the number of serial M&A, the degree of digital transformation, and the enterprises’ TFP. This paper takes China’s serial M&A of listed companies from 2010 to 2019 as a sample to explore the impact of the number of serial M&As on the enterprises’ TFP and the mechanism of the degree of digital transformation in the two. The study found that:

Firstly, the number of serial M&A has a significant inverse U-shaped relationship with the enterprises’ TFP. With the increase in the number of serial M&A, the development quality of enterprises shows a trend of first rising and then declining. This indicates that excessive serial M&A may hurt the enterprises’ TFP, thus reminding enterprise managers that they need to implement M&A transactions reasonably and control the number of serial M&A to achieve the best results.

Secondly, the degree of digital transformation of enterprises plays an intermediary role in the inverted U-shaped relationship between the number of serial M&A and the enterprises’ TFP. Digital transformation can alleviate the negative impact of excessive serial M&A on the enterprises’ TFP to a certain extent, thus providing an important development strategy for enterprises. Therefore, enterprise managers should make full use of the positive impact of digital transformation on the TFP of serial M&A.

In addition, heterogeneity analysis shows that in the case of non-state-owned property rights, small enterprise scale, and high enterprise business environment index, the number of serial M&A has a more significant mediating effect on the inverted "U" shape of enterprise development quality and the degree of digital transformation. This suggests that the government regulatory authorities should formulate targeted policies for different types of enterprises to support and monitor the serial M&A of enterprises, and establish a legal system suitable for the digital transformation and development of enterprises, to promote the high-quality development of serial M&A.

### 6.2. Research implications

#### 6.2.1. Theoretical implication

Existing studies have focused on the impact of a single M&A on TFP, but have not yet reached a unified conclusion. In terms of the relationship between M&A, digital transformation and TFP, the existing studies focus on the impact of a single M&A on TFP and digital transformation on TFP and seldom put them into a research framework. Digital transformation is used as a mediating variable to test the action mechanism of serial M&A on TFP. Existing studies have focused on the significant impact of the nature of property rights, firm size and business environment on corporate mergers and acquisitions. In this study, in the indirect impact of serial M&A on TFP through digital transformation, we have also found differences in the impact of different property rights, firm size and business environment index.

In this study, we have established a theoretical framework to explore how and under what conditions serial M&A affect TFP. This study contributes to the existing research in three aspects. Firstly, the impact of serial M&A on the inverted U-shaped relationship of TFP helps to reconcile the existing contradictory views [1, 2, 25, 33, 49, 50]. On the one hand, it is different from the simple linear relationship between M&A and TFP in previous studies [22–24, 33], we have found that the effect of M&A on TFP is inverted U-shaped. There is a turning point, which means that a medium-level M&A can bring optimal TFP to the enterprises. On the other hand, taking into account the potential cost factors of serial M&A, our framework provides a holistic perspective, which helps to extend the focus of the study to optimise enterprises’ serial M&A decision-making [38, 46]. The reason is that excessive pursuit of M&A can easily lead to misadaptation with the existing infrastructure and management systems [34, 35]. Due to the differences in corporate culture, the reorganization of human resources and the limitation of managers’ ability, it is difficult to achieve efficient resource integration after serial M&A exceed a certain number, so it is not conducive to the promotion of TFP.

Secondly, we study the influence mechanism of how serial M&A affects TFP from the perspective of digital transformation, contributing to the research of TFP. Previous researchers have discussed the impact of M&A on TFP [1, 25, 50], or addressed the impact of digital transformation on TFP [16–19]. In contrast, the results of this study reveal the mediating role of digital transformation: serial M&A indirectly affect the enterprises’ TFP through digital transformation, which provides new empirical evidence for the research results of most serial M&A and digital transformation literature. The results of this study explain to some extent why the moderate serial M&A is optimal for the enterprises’ TFP, and provide the possible reasons why serial M&A are difficult to achieve expectations.

Thirdly, we conduct heterogeneity analysis from the perspective of property rights, firm size and business environment index. In terms of the nature of corporate property rights, previous studies have found the impact of property rights on cross-border M&A with high valuation [86], disclosure restraint mechanism of target enterprises [87], and innovation performance of M&A enterprises [60]. From the perspective of firm size, previous studies have found that even small changes in firm size will have a significant impact on the model of study [79]. Vijh and Yang (2013) have found the impact of firm size on the possibility of M&A [88]. Gabaix, Landier and Sauvagnat (2014) have found a close relationship between CEO compensation and firm size [89]. Zhang, Song and Liu (2023) have found that different firm sizes have different paths for serial M&A to affect innovation performance [60]. Our study results show that the effect of serial M&A on TFP indirectly through digital transformation is more obvious for smaller enterprises. In terms of the external environment faced by enterprises, Bialek and Weichenrieder (2021) have found that environmental regulation reduces greenfield investment in polluting industries, but has little impact on the number of M&A [90]. Li, Su and Wang (2022) have found the impact of economic policy uncertainty on M&A behaviour and M&A performance [91]. The results of this study show that the business environment index has an impact on the TFP of serial M&A, which provides micro-level empirical evidence for a deep understanding of how business environment optimization affects TFP.

#### 6.2.2. Practical implication

From the perspective of practice, this study can provide valuable empirical evidence and policy implications for enterprise decision-makers. First of all, the number of serial M&A has a positive impact on the enterprises’ TFP, but it also reveals that excessive serial M&A will damage the enterprises’ TFP. This means that enterprise decision-makers need to carefully consider M&A decisions, reasonably implement M&A transactions, and control the number of serial M&A to achieve the best results. Secondly, our study finds that digital transformation plays a mediating role between serial M&A and corporate TFP. This means that enterprise decision-makers should make full use of the positive impact of digital transformation on the TFP of serial M&A enterprises. Especially for enterprises with non-state-owned property rights, relatively small enterprises and high business environment index, it is more necessary to make use of digital transformation to give full play to the left positive impact of serial M&A on the TPF U-shaped relationship.

At the same time, government regulatory departments can also improve M&A policies according to our research results, actively guide enterprises to carry out serial M&A, reduce the adverse effects of excessive M&A, and promote the enterprises’ TFP. Government regulators can introduce policies to guide and promote the enterprises’ digital transformation, to enhance the positive impact of serial M&A on the left side of TFP in the U-shaped curve relationship. For enterprises with different property rights, firm size and business environment index, government regulators should implement differential management and more stringent M&A controls should be imposed on state-owned enterprises, larger enterprises and enterprises with lower business environment index. At the same time, we should reasonably support and monitor the serial M&A activities of enterprises, establish a legal system suitable for the development of digital transformation, and promote the high-quality development of serial M&A enterprises.

### 6.3. Research limitations and prospects

There are some limitations to the study. Firstly, the sample selection is still limited to listed companies in China and does not cover the data of enterprises in other countries or regions, so the universality of the transnational scope needs to be further discussed. Secondly, the measurement indicators of digital transformation and the definition of serial M&A may also affect the research results. Future research can further optimize the indicator system. In addition, this study does not consider other factors that may affect the enterprises’ TFP, such as changes in the macroeconomic environment, which can be conducted more comprehensively in the future. Future research can further deepen the research on the influence mechanism of digital transformation on the M&A enterprises’ TFP, explore the differences in different industrial and national backgrounds, and provide more accurate suggestions for corporate decision-making and policy-making based on empirical data.

### 6.4. Conclusion

This study highlights the mediating role of digital transformation in the inverted U-shaped relationship between serial M&A and enterprises’ TFP. The accumulation of M&A experience brings synergies to serial M&A enterprises, which helps to optimize resource allocation, reduce redundancy, and improve efficiency, thereby increasing enterprises’ TFP. However, as the number of serial M&A increases, enterprises have M&A inertia due to the accumulation of experience and knowledge, and experience and knowledge gradually depreciate, and the difficulty of M&A integration and organizational inertia impede the improvement of enterprises’ TFP in serial M&A. Digital transformation improves the overall operational efficiency of enterprises through digital technology, which in turn promotes the improvement of enterprises’ TFP. The new technological resources acquired through serial M&A are reorganized with the enterprise’s inherent resources, providing a knowledge and technology foundation for the enterprise to enhance the degree of digital transformation. However, as the number of serial M&A increases, the driving effect of serial M&A on enterprise digital transformation declines, inhibiting the improvement of enterprises’ TFP.

## Supporting information

S1 Data Research data is available in the supporting information. (XLSX)
